# Thyroidectomy in thyrotoxic crisis: is a euthyroid state required? A case series

**DOI:** 10.1093/jscr/rjaf765

**Published:** 2025-09-30

**Authors:** Guillermo Guzmán, Andres O Garcia, Oriana A Valderrama, Stefania Calderón, Julián A Molano, William Victoria, Luis F Tintinago, Juan L O Giraldo

**Affiliations:** Departamento de Medicina Interna, Endocrinología, Fundación Valle del Lili, Cra 98 No. 18 - 49, Cali 760032, Colombia; Departamento de Medicina Interna, Endocrinología, Fundación Valle del Lili, Cra 98 No. 18 - 49, Cali 760032, Colombia; Departamento Medicina, Universidad Icesi, Facultad Salud, Calle 18 No. 122 -135, Cali 760032, Colombia; Centro de Investigaciones Clínicas, Fundación Valle del Lili, Cra 98 No. 18 - 49, Cali 760032, Colombia; Centro de Investigaciones Clínicas, Fundación Valle del Lili, Cra 98 No. 18 - 49, Cali 760032, Colombia; Departamento de Cirugía Cabeza y Cuello, Fundación Valle del Lili, Cra 98 No. 18 - 49, Cali 760032, Colombia; Departamento de Cirugía Cabeza y Cuello, Fundación Valle del Lili, Cra 98 No. 18 - 49, Cali 760032, Colombia; Departamento de Cirugía Cabeza y Cuello, Fundación Valle del Lili, Cra 98 No. 18 - 49, Cali 760032, Colombia; Centro de Investigaciones Clínicas, Fundación Valle del Lili, Cra 98 No. 18 - 49, Cali 760032, Colombia

**Keywords:** thyroid storm, thyrotoxicosis, thyroidectomy, perioperative management, beta-blockers, case series

## Abstract

Thyrotoxic crisis is a life-threatening emergency where refractory cases require thyroidectomy. Achieving preoperative euthyroidism is difficult, and optimal surgical timing is debated. This study assessed outcomes of early thyroidectomy regardless of preoperative thyroid levels. A retrospective review included patients ≥18 years (2011–2020) meeting thyroid storm criteria (Japanese Thyroid Association and Burch-Wartofsky >25). Twelve women (median age 36) were included, primarily for medical treatment failure (83.3%). All received beta-blockers; median heart rate decreased from 110 to 76.5 bpm. All underwent total thyroidectomy with 100% parathyroid preservation. Complications included transient hypocalcemia and hypoparathyroidism (41.67% each). There was no mortality, thromboembolism, or need for renal replacement. Early thyroidectomy is a viable option for medically refractory thyrotoxic crisis. Strict heart rate control appears more critical than achieving preoperative euthyroidism, supporting its safety and feasibility.

## Introduction

Hyperthyroidism is characterized by the irregular synthesis and secretion of hormones from the thyroid gland, resulting in increased serum blood levels of thyroid hormones [1, 2]. Thyrotoxicosis refers to the clinical implications of an excess of thyroid hormones in the blood, and thyrotoxic crisis is the most severe expression of thyrotoxicosis. It is a rare, life-threatening endocrine condition with a reported mortality of 10%–30% and must be diagnosed and treated at the early as possible [1, 3, 4].

Medical treatment includes supportive measures and control of heart rate with beta-adrenergic blockers, glucocorticoids, and antithyroid drugs (thionamides). In thyrotoxic crisis, admission to an intensive care unit is necessary, along with early identification of precipitating factors [1, 5]. In patients with a multisystemic compromise who require rapid restoration of normal thyroid hormone levels (i.e. euthyroidism), radioactive iodine is contraindicated, and thyroidectomy becomes the treatment of choice. However, these patients typically require preoperative treatment for thyrotoxicosis, and almost all require therapeutic plasmapheresis as a bridge to surgery is an attempt to achieve a euthyroid state [6, 7]. In practice, however, this is challenging, and thyroidectomy is often performed despite persistent thyrotoxicosis. Determining the optimal timing for surgery remains a clinical challenge.

There is limited information on the outcomes of early thyroidectomy in patients with thyrotoxic crisis. The aim of the study was to describe the outcomes of thyroidectomy in patients with thyrotoxic crisis who underwent surgery without achieving a euthyroid state.

## Material and methods

### Study design and population

This was a retrospective single-center study covering the period from 1 January 2011 to 31 December 2020. All patients were identified through the electronic medical record system using the following International Classification of Diseases, 10th Revision (ICD-10) codes for thyrotoxicosis: E05.5 (Thyroid crisis or storm), E05.8 (Other thyrotoxicosis), E05.9 (Thyrotoxicosis, unspecified), E05 (Thyrotoxicosis [Hyperthyroidism]), E05.0 (Thyrotoxicosis with diffuse goiter), E05.1 (Thyrotoxicosis with single toxic thyroid nodule), and E05.2 (Thyrotoxicosis with toxic multinodular goiter).

### Participants and data collection

Participants aged ≥18 years with a diagnosis of thyrotoxic crisis at admission who subsequently underwent thyroidectomy were included. After reviewing electronic medical records, only those who met the criteria for suspected or definite thyroid storm according to the Japanese Thyroid Association (JTA) and with a Burch-Wartofsky scale score ˃25 were selected. All patients had a target preoperative heart rate of 90 bpm in accordance with local protocols. Pregnant patients were excluded.

Demographic data collected included age, sex, and medical history. Regarding hyperthyroidism, we recorded time since diagnosis, underlying cause, presence of orbitopathy, and prior treatments. We also documented precipitating factors and clinical variables at presentation to the emergency department and during hospitalization.

Laboratory data collected during hospitalization included preoperative thyroid function test—thyroid-stimulating hormone (TSH; reference range 0.27–4.2 mIU/L) and free thyroxine (free T4; reference range 0.92–1.68 ng/dL) measured by electrochemiluminescence—as well as renal function, electrolyte levels, arterial blood gasses, and coagulation profiles. Additionally, echocardiographic findings and perioperative thyroidectomy variables were analyzed. Postoperative outcomes included need for ICU admission, total hospital stay, pulmonary thromboembolism, recurrent laryngeal nerve injury, hypothyroidism, hypoparathyroidism, need for renal replacement therapy, and mortality.

### Statistical analysis

Data analysis was performed using STATA version 15.0. Qualitative variables were summarized as frequencies and proportions. The Shapiro–Wilk test was used to assess the normality of quantitative variables. Normally distributed variables were expressed as mean ± standard deviation, and non-normally distributed variables were expressed as median and interquartile range (IQR).

### Ethical aspects

The study protocol was reviewed and approved by the institutional Review Board and Ethics Committee (meeting No. 459 on December 01–2021). Written informed consent was obtained from each patient after a full explanation of the purpose and nature of all procedures performed.

## Results

**Figure 1:**
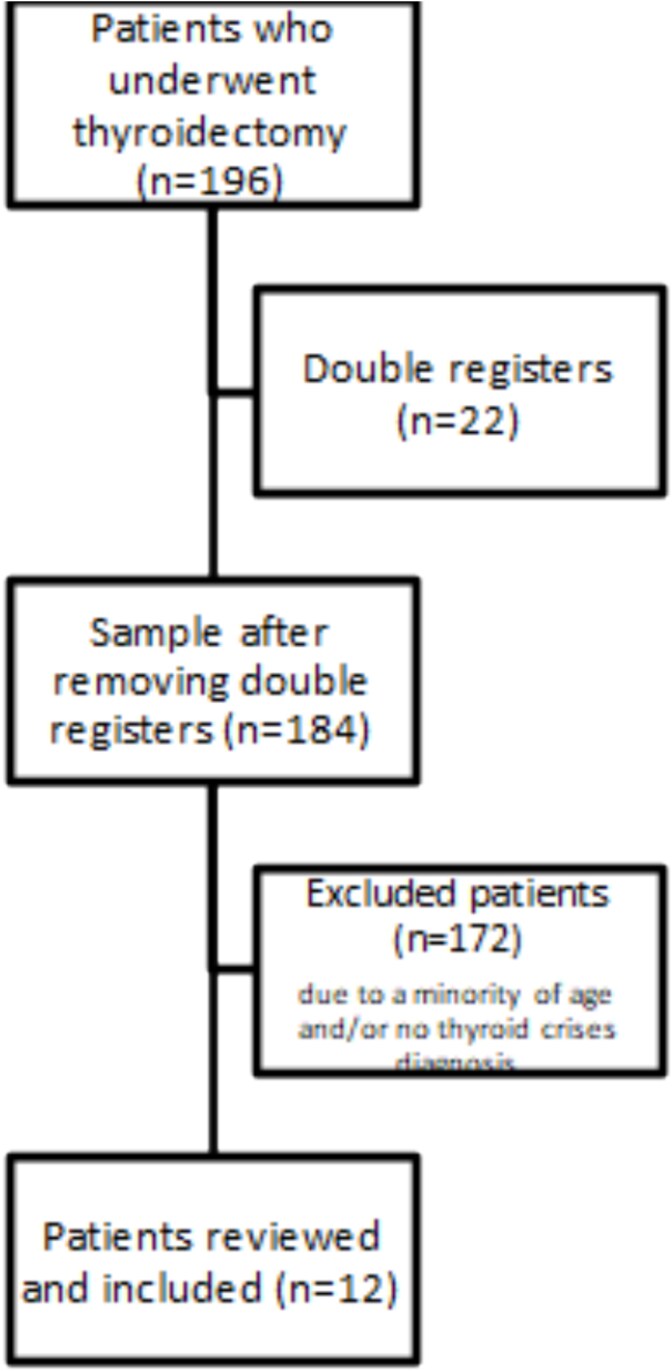
The selection process of patients included in the study.

Of the 196 participants who underwent thyroidectomy and were initially eligible for the study, 172 were excluded, leaving 12 patients for analysis (Fig. 1). All were women, with a median age of 36 years (IQR, 31.5–42.5). A prior diagnosis of hyperthyroidism was present in 66.6% of patients, with a median disease duration of 3 years (IQR, 2–5). Graves’ disease was the most common etiology (75%), followed by toxic multinodular goiter (25%). Only 25% of patients had been receiving antithyroid therapy prior to hospitalization, all of them with methimazole. At presentation, the median heart rate was 110 bpm (IQR, 95.5–115), and median preoperative free T4 and TSH levels were 6.205 ng/dL (IQR, 3.08–7.77) and 0.005 μUI/mL (IQR, 0.005–0.005), respectively (Table 1). Serum T3 levels were not measured due to the unavailability of the assay at our institution.

**Table 1:** General characteristics of patients with thyrotoxic crisis underwent thyroidectomy.

**Characteristics**	**General, n = 12**
Age (years)^a^	36 (31.5–42.5)
Sex: women, n (%)	12 (100)
BMI^a^	24.75 (25.57–27.6)
Normal weight < 25, n (%)	5 (41.6)
Overweight 25–29.9, n (%)	4 (33.3)
Obese >30, n (%)	1 (8.3)
Medical history, n (%)	
Hypertension	3 (25.0)
Asthma	0 (0)
Atrial fibrillation	1 (8.3)
Heart failure	2 (16.6)
Hyperthyroidism, n (%)	
Previous diagnosis	8 (66.6)
Diagnosis time, in years^a^	3 (2–5)
Orbitopathy, n (%)	5 (45.4)
Hyperthyroidism cause, n (%)	
Graves’ disease	9 (75.0)
Toxic multinodular goiter	3 (25.0)
Prior antithyroid treatment, n (%)	3 (25.0)
Methimazole	3 (25.0)
Heart rate at admission bpm^a^	110 (95.5–115)
Biochemical data	
Free Thyroxine ng/dL^a^	6.205 (3.08–7.77)
TSH uUI/mL^a^	0.005 (0.005–0.005)
PTH pg/mL^a^	14.6 (5.3–29.81)
Frequent symptoms, n (%)	
Vomiting	4 (33.3)
Systemic edema	4 (33.3)
Pulmonary edema	2 (16.6)
Acute hepatic failure	2 (16.6)
Predisposing factor, n (%)	
Acute disease	9 (75)
Not identified	1 (8.3)
Withdrawal of antithyroid medications	2 (16.6)
Echocardiogram, n (%)	7 (58.33)
LVEF^a^	54.5 (39–60)
Left atrial volume^a^	29 (22–45)
Pulmonary artery systolic pressure^a^	44 (35–49)
Mitral valve disease, n (%)	5 (41.67)

Values are reported as absolute numbers (percentages).

^a^Reported as median (IQR: interquartile range, BMI: body mass index, TSH: Thyrotropin, PTH: Parathyroid hormone).

In the 24 h before surgery, the median mean arterial pressure was 91.5 mmHg (IQR, 84.5–103.5), and the median heart rate decreased to 76.5 bpm (IQR, 71–81.5). All patients received beta-blocker therapy, with a median total daily dose of 126.5 mg (IQR, 84–342) for a median duration of 9.5 days (IQR, 6.5–15.5). Additionally, all patients received methimazole 120 mg daily and glucocorticoids, and 91.67% were treated with cholestyramine. Only one patient (8.33%) required plasmapheresis as a bridge to surgery (Table 2). Only one patient received iodine (Lugol’s solution) as part of the thyrotoxicosis management, due to limited availability.

**Table 2:** Hospital course of patients before thyroidectomy.

**Characteristics**	**General, n = 12**
Heart rate (24 h before) bpm^a^	76.5 (71–81.5)
Free Thyroxine (24 h before) ng/dL^a^	4.295 (2.705–5.795)
Beta-blocker Treatment	
Propranolol, n (%)	12 (100)
Total daily dose^a^	126.5 (84–342)
Total days of propranolol^a^	9.5 (6.5–15.5)
Methimazole, n (%)	12 (100)
Iodine therapy, n (%)	1 (8.33)
Glucocorticoids, n (%)	12 (100)
Plasmapheresis, n (%)	1 (8.33)
Cholestyramine, n (%)	11 (91.67)

Values are reported as absolute numbers (percentages).

^a^Reported as median (IQR: interquartile range, BMI: body mass index, TSH: Thyrotropin, PTH: Parathyroid hormone).

We applied the Japanese Thyroid Association (JTA) criteria for thyroid storm to evaluate the agreement with the Burch-Wartofsky Score. All patients (100%) met the criteria for suspected or definite thyroid storm. Based on the Burch-Wartofsky score, 55.56% had a score > 45, indicating definite thyroid storm, while 44.4% scored between 25 and 45, suggesting an impending thyroid crisis (Table 3).

**Table 3:** Diagnosis of thyroid storm by Japanese thyroid association criteria and the corresponding Burch–Wartofsky score and ASA score.

**Japanese Thyroid Association Criteria**	**Burch–Wartofsky Score** ^**c**^		
	**<25**	**25–45**	**>45**
TS1 Definite^*^^,^^a^	0	4 (44.4)	5 (55.56%)
TS2 Suspected^*^^,^^b^	0	3 (100)	0 (100%)

Values are reported as absolute number (percentages). ^*^Reported as median (IQR interquartile range).

^a^Diagnostic criteria for Definite: thyrotoxicosis and at least one central nervous system (CNS) manifestation plus one or more of other manifestations (fever of tachycardiac or congestive heart failure or gastrointestinal/hepatic), or three or more of the manifestation just listed other than CNS manifestation [3].

^b^Diagnostic criteria for ‘suspected’: thyrotoxicosis plus two of the manifestation listed above, excluding CNS manifestation [3].

^c^According to Burch Wartosky Point Scale, total score or > 45 is likely thyroid storm; 22 = 444 is suggestive of an impending thyroid storm; and < 25 is unlikely to represent a thyroid storm [11].

All patients underwent total thyroidectomy with parathyroid gland preservation. The primary indication for surgery was medical treatment failure in 83.33% of cases, followed by patient preference (8.33%) and thyroid cancer (8.33%). Postoperative complication included transient hypoparathyroidism (41.67%) and hypocalcemia (41.67%). One patient (8.33%) required vasopressor support and mechanical ventilation. No cases of pulmonary thromboembolism or renal replacement therapy were reported. The median ICU stay was 16 days (IQR, 11.5–23.5), and there was no deaths (Table 4).

**Table 4:** Thyroidectomy outcomes.

**Characteristics**	**General, n = 12**
Reason for choosing operation	
Failed medical therapy	10 (83.33)
Patient preference	1 (8.33)
Cancer	1 (8.33)
ASA Grade	
ASA I, n (%)	0 (0)
ASA II, n (%)	3 (25)
ASA III, n (%)	9 (75)
Thyroidectomy	
Total, n (%)	12 (100)
Partial, n (%)	0 (0)
Parathyroid glands preservation, n (%)	12 (100)
Surgery time in minutes	113 (82.5–152)
Complications	
Post-operative hypocalcemia, n (%)	5 (41.67)
Post-operative transitory hypoparathyroidism, n (%)	5 (41.67)
Post-operative hypothyroidism, n (%)	12 (100)
Mean arterial pressure (24 h post-surgery)^a^	89 (80.5–99.5)
Heart rate (24 h post-surgery)^a^	82.5 (72–94.5)
Pulmonary thromboembolism, n (%)	0(0)
Renal replacement therapy, n (%)	0 (0)
Vasopressor requirement, n (%)	1(8.33)
Mechanical ventilation, n (%)	1 (8.33)
Mortality, n (%)	0 (0)

Values reported as absolute numbers (percentage).

^a^Reported as median. RLN: Recurrent Laryngeal Nerve, ASA: American Society of Anesthesiologists.

## Discussion

Our case series highlights the outcomes of early thyroidectomy in patients with thyrotoxic crisis, providing valuable insights into the management of this life-threatening condition. Thyrotoxic crisis is an endocrine emergency—a severe manifestation of thyrotoxicosis—characterized by multisystem dysfunction involving the cardiovascular, thermoregulatory, gastrointestinal, and central nervous systems, with a high associated mortality of 10%–30% [8, 9]. Furthermore, it is a rare condition with an incidence of 0.55 cases per 100 000 person-years in Taiwan and 4.8–5.6 cases per 100 000 hospitalized patients annually in the United States [10].

Surgical management is considered a last resort for thyrotoxic crisis because of the risks associated with surgery [10–13]. In our cohort the principal indication for thyroidectomy was medical treatment failure (83.3%), consistent with current recommendations for refractory thyrotoxic crisis [11, 12]. Notably, we performed surgery regardless of FT4 levels, prioritizing strict heart rate control with beta-blockers. Although the goal is to achieve a euthyroid state before surgery, this is often challenging in practice. In some instances, multiple plasmapheresis sessions are required, which carry a potential risk of complications [14, 15]. However, in certain cases, that state cannot be achieved [16, 17]. In our series, only one patient required plasmapheresis and still did not reach euthyroidism before surgery.

Prioritizing heart rate control may be a pragmatic approach that leads to favorable clinical outcomes in complex cases. The use of beta-blockers in non-cardiac surgery has been controversial [18–20]. Recent evidence suggests that beta-blocker use is not associated with a reduction in cardiac complications such as perioperative myocardial infarction, cardiac injury, or major adverse cardiac events [21]. However, in the context of perioperative tachyarrhythmia—especially in thyrotoxic crisis—tight heart rate control may be of greater clinical importance. Clinical trials have shown that achieving optimal perioperative heart rate control is associated with a lower incidence of postoperative myocardial infarction, emphasizing the cardio protective role of this strategy [22]. It is not merely the use of beta-blockers that matters, but rather the achievement of adequate heart rate control, which is typically elevated in patients experiencing thyrotoxic crisis. In our study, all patients had heart rates <90 bpm on the day before surgery. Additionally, the use of propranolol—a non-selective β-adrenergic antagonist with a half-life of ⁓3–6 h—offers dual benefits: effective control of tachycardia and reduction of peripheral conversion of T4 to the more active T3, thereby attenuating systemic thyrotoxic symptoms. In our cohort, propranolol was chosen over other beta-blockers due to its combined cardiac and metabolic effects, local availability, and clinician experience. Alternative agents such as esmolol (ultra-short-acting, intravenous) or metoprolol (cardio selective, longer-acting) may be considered in specific contexts, such as hemodynamic instability or reactive airway disease.

Thyrotoxic crisis remains a challenging diagnosis, as its symptoms often overlap with those of other acute conditions. Diagnostic criteria have therefore been developed to improve accuracy [9]. In 1993 Burch and Wartofsky proposed a scoring system that stratifies likelihood of thyroid storm based on clinical parameters; a score ≥ 45 is highly suggestive of thyroid storm [23]. The Japanese Thyroid Association (JTA) introduced new diagnostic criteria based on nationwide surveys, classifying patients as TS1 (definite) or TS2 (suspected) thyroid storm [14]. To improve diagnostic precision, the use of both scoring systems in parallel has been suggested [14]. In our study, all patients met JTA criteria for suspected or definite thyroid storm. Among those classified as definite by JTA, 55.6% had a Burch–Wartofsky score >45, while 44.4% scored between 25 and 45, indicating an impending crisis. Although prior studies have shown general concordance between these systems, discrepancies can occur. These findings highlight the variability inherent in diagnosis and reinforce the value of combining both tools for more accurate clinical assessment [3, 14].

All patients underwent total thyroidectomy with parathyroid preservation. Postoperative complications were limited to with transient hypocalcemia and hypoparathyroidism observed in 41.67% of patients, rates consistent with published data on thyroid surgery [11, 24]. No patients developed thromboembolism or required renal replacement therapy. The median ICU stay was 16 days.

Mortality among patients with thyrotoxic crisis undergoing thyroidectomy has been reported to be ⁓10% [11, 24]. Factors associated with increased mortality include age > 60 years, central nervous system dysfunction at admission, omission of medical treatment, and the need for mechanical ventilation or plasma exchange [25]. Our cohort did not had no in-hospital deaths, possibly due to the younger median age, aggressive perioperative management, absence of severe organ failure at admission, and the expertise of both surgical and critical care teams.

A potential limitation in interpreting our results was the unavailability of free T3 measurements. Nevertheless, given the severity of clinical presentation and the markedly elevated free T4 levels, it is unlikely that free T3 values would have been within the normal range.

The main limitation of our study is its retrospective design and the small sample size. Therefore, these findings should be interpreted within the context of observational research. However, to our knowledge, this is the largest case series describing thyrotoxic crisis patients undergoing thyroidectomy. Based on our experience, early thyroidectomy may be considered a treatment option for patients refractory to medical therapy, with the goal of reducing mortality—even in the absence of a euthyroid state—by prioritizing strict heart rate control.

## Conflict of interest statement

The authors declare no conflict of interest.

## Funding

This research did not receive any specific grant from any funding agency in the public, commercial, or not-for-profit sector.

## Ethical aspects

The study protocol was reviewed and approved by the institutional review board and ethics in their committee meeting No. 459 on December 01–2021. Consent has been obtained from each patient or subject after full explanation of the purpose and nature of all procedures used.

## References

[1] Angell TE, Lechner MG, Nguyen CT, et al. Clinical features and hospital outcomes in thyroid storm: a retrospective cohort study. J Clin Endocrinol Metab 2015;100:451–9. 10.1210/jc.2014-2850 25343237

[2] Mercado, Cruz E, Fernando García Cubría C, Arellano Tejeda A, et al. Tormenta tiroidea, una emergencia endocrina Presentación de un caso y revisión de la literatura. *Rev Fac Med* 2017;60:27–36.

[3] Akamizu T, Satoh T, Isozaki O, et al. Diagnostic criteria, clinical features, and incidence of thyroid storm based on nationwide surveys. Thyroid. 2012;22:661–79. 10.1089/thy.2011.0334 22690898 PMC3387770

[4] Guzmán G, Arango LG, Cañas A, et al. Tirotoxicosis severa y tormenta tiroidea: una serie de casos. Rev argent. Endocrinol Metab 2020;57:26–31.

[5] Bourcier S, Coutrot M, Kimmoun A, et al. Thyroid storm in the ICU: a retrospective multicenter study. Crit Care Med 2020;48:83–90. 10.1097/CCM.0000000000004078 31714398

[6] de Mul N, Damstra J, Nieveen van Dijkum EJM, et al. Risk of perioperative thyroid storm in hyperthyroid patients: a systematic review. Br J Anaesth 2021;127:879–89. 10.1016/j.bja.2021.06.043 34389171

[7] Guzmán GGE, Bautista RDF, Arango VLG. Therapeutic plasmapheresis in patients with thyrotoxicosis. Report of two cases. Rheumatol Curr Res 2015;05:1–4. 10.4172/2161-1149.1000162

[8] Idrose AM . Acute and emergency care for thyrotoxicosis and thyroid storm. Acute Med Surg 2015;2:147–57. 10.1002/ams2.104 29123713 PMC5667251

[9] Maguy C, Shanika S, Kabaker AS. Thyroid storm: an updated review. Sage J 2013;30:131–40. 10.1177/0885066613498053 23920160

[10] Kornelius E, Chang KL, Yang YS, et al. Epidemiology and factors associated with mortality of thyroid storm in Taiwan: a nationwide population-based study. Intern Emerg Med 2021;16:601–7. 10.1007/s11739-020-02445-6 32676839

[11] Alahmad M, Al-Sulaiti M, Abdelrahman H, et al. Extracorporeal membrane oxygenation support and total thyroidectomy in patients with refractory thyroid storm: case series and literature review. J Surg Case Rep 2022;2022:rjac131. 10.1093/jscr/rjac131 PMC9113021 35592452

[12] Scholz GH, Hagemann E, Arkenau C, et al. Is there a place for thyroidectomy in older patients with Thyrotoxic storm and cardiorespiratory failure? Thyroid®. 2003;13:933–40. 10.1089/105072503322511337 14611702

[13] Aulet RM, Wein RO, Siegel RD. Surgical management of an atypical presentation of a thyroid storm. Int. J Endocrinol Metab 2014;12:e13539. 10.5812/ijem.13539 PMC3997950 24782903

[14] Satoh T, Isozaki O, Suzuki A, et al. 2016 guidelines for the management of thyroid storm from the Japan Thyroid Association and Japan Endocrine Society (first edition). Endocr J 2016;63:1025–64. 10.1507/endocrj.EJ16-0336 27746415

[15] Koh H, Kaushik M, Loh JK, et al. Plasma exchange and early thyroidectomy in thyroid storm requiring extracorporeal membrane oxygenation. Endocrinol Diabetes Metab Case Rep 2019;2019:1–6. 10.1530/EDM-19-0051 PMC6685092 31352696

[16] Simsir IY, Ozdemir M, Duman S, et al. Therapeutic plasmapheresis in thyrotoxic patients. Endocrine 2018;62:144–8. 10.1007/s12020-018-1661-x 29968224

[17] Ezer A, Caliskan K, Parlakgumus A, et al. Preoperative therapeutic plasma exchange in patients with thyrotoxicosis. J Clin Apher 2009;24:111–4. 10.1002/jca.20200 19484727

[18] POISE Study Group, Devereaux PJ, Yang H. Effects of extended-release metoprolol succinate in patients undergoing non-cardiac surgery (POISE trial): a randomised controlled trial. Lancet 2008;371:1839–47. 18479744 10.1016/S0140-6736(08)60601-7

[19] Karam D, Arora R. Perioperative b-blockers in patients undergoing noncardiac surgery-scientific misconduct and clinical guidelines. Am J Ther 2017;24:e435–41. 10.1097/MJT.0000000000000548. 28092285

[20] Blessberger H, Kammler J, Domanovits H, et al. Perioperative beta-blockers for preventing surgery-related mortality and morbidity (review). Cochrane Database Syst Rev 2014;9:CD004476. 10.1002/14651858.CD004476.pub2. 25233038

[21] Glarner N, Puelacher C, Gualandro DM, et al. Association of preoperative beta-blocker use and cardiac complications after major noncardiac surgery: a prospective cohort study. Br J Anaesth 2024;132:1194–203. 10.1016/j.bja.2024.02.023 38627137

[22] Beattie WS, Wijeysundera DN, Karkouti K, et al. Does tight heart rate control improve beta-blocker efficacy? An updated analysis of the noncardiac surgical randomized trials. Anesth Analg 2008;106:1039–48. 10.1213/ane.0b013e318163f6a9 18349171

[23] Burch HB, Wartofsky L. Life-threatening thyrotoxicosis: thyroid storm. Endocrinol Metab Clin North Am 1993;22:263–77. 10.1016/S0889-8529(18)30165-8 8325286

[24] Uchida N, Suda T, Ishiguro K. Thyroidectomy in a patient with thyroid storm: report of a case. Surg Today 2015;45:110–4. 10.1007/s00595-013-0754-7 24132684

[25] Ono Y, Ono S, Yasunaga H, et al. Factors associated with mortality of thyroid storm analysis using a national inpatient database in Japan. Medicine (United States) 2016;95:e2848. 10.1097/MD.0000000000002848 PMC4998648 26886648

